# Lighting Up *Clostridium Difficile*: Reporting Gene Expression Using Fluorescent Lov Domains

**DOI:** 10.1038/srep23463

**Published:** 2016-03-21

**Authors:** Anthony M. Buckley, Caitlin Jukes, Denise Candlish, June J. Irvine, Janice Spencer, Robert P. Fagan, Andrew J. Roe, John M. Christie, Neil F. Fairweather, Gillian R. Douce

**Affiliations:** 1 Institute of Infection, Immunity and Inflammation, College of Medical, Veterinary & Life Sciences, University of Glasgow, Glasgow G12 8TA U.K; 2 Department of Molecular Biology and Biotechnology, University of Sheffield, Western Bank, Sheffield S10 2TN U.K; 3 Division of Cell and Molecular Biology, Centre for Molecular Microbiology and Infection, Imperial College London, London SW7 2AZ U.K; 4 Institute of Molecular Cell and Systems Biology, College of Medical, Veterinary and Life Sciences, University of Glasgow, Glasgow G12 8TA U.K

## Abstract

The uses of fluorescent reporters derived from green fluorescent protein have proved invaluable for the visualisation of biological processes in bacteria grown under aerobic conditions. However, their requirement for oxygen has limited their application in obligate anaerobes such as *Clostridium difficile*. Fluorescent proteins derived from Light, Oxygen or Voltage sensing (LOV) domains have been shown to bridge this limitation, but their utility as translational fusions to monitor protein expression and localisation in a strict anaerobic bacterium has not been reported. Here we demonstrate the utility of phiLOV in three species of *Clostridium* and its application as a marker of real-time protein translation and dynamics through genetic fusion with the cell division protein, FtsZ. Time lapse microscopy of dividing cells suggests that Z ring assembly arises through the extension of the FtsZ arc starting from one point on the circumference. Furthermore, through incorporation of phiLOV into the flagella subunit, FliC, we show the potential of bacterial LOV-based fusion proteins to be successfully exported to the extracellular environment.

*Clostridium difficile* is a pathogen of major significance worldwide, being recognised as the leading cause of antibiotic associated diarrhoea in the nosocomial setting. In the U.S. increased rates of *C. difficile* infection (CDI) and relapse, seen in up to 20.9% of CDI these cases^1^, incur annual health care costs in excess of $4.8 billion^2^. Consequently, there is an urgent need to understand the pathogenic traits of this organism and the influence of expression of specific genes during infection. To date, most research has centred on the study of the two major toxins A and B (TcdA & TcdB) whose production and activity are responsible for the majority of symptoms observed during CDI^3^,^4^. Whilst experimental vaccines based on these toxins reduce or eliminate symptoms^5^ they do not prevent germination, colonisation and sporulation of the bacteria, which are all essential steps in transmission from host to host. Greater understanding of these processes is essential if we are to identify new targets for future prophylactic or therapeutic treatments.

One factor limiting insight into the molecular analysis of this pathogen is the inability to visualise and monitor ongoing cellular processes. Fluorophores, derived from green fluorescent protein (GFP) have been used successfully in other species^6^, but require molecular oxygen for fluorescence maturation, thus preventing their use in anaerobic systems. In addition, many *Clostridium* species generate a green autofluorescence when excited with blue/ultraviolet (UV) light^7^ making visualisation with green fluorescent reporters problematic. However, advances in *C. difficile* bioimaging are beginning to occur, with the oxygen-dependent fluorescent proteins [Cyan Fluorescent Protein (CFP) and mCherry] used to characterise the cellular localisation of MdlA/B/C & ZapA in dividing bacteria^7^,^8^. In addition, tags based on O-6-methylguanine-DNA methyltransferase (SNAP-tags) have been used successfully to investigate protein localisation^9^,^10^. These elegant studies have significantly extended our understanding of the temporal and spatial interplay between the RNA polymerase sigma subunits during sporulation. However, due to oxygen requirements or cell toxicity of substrates used, they are unsuited for monitoring real time protein dynamics in anaerobic environments. In contrast, small flavin-based photosensory modules such as Light, Oxygen or Voltage sensing (LOV) domains have shown promising potential as real time probes in oxygen depleted situations^11^,^12^,^13^,^14^,^15^.

LOV domains were first identified in plants associated with the blue-light receptor kinases^16^ (phototropins) and bind flavin mononucleotide (FMN) as a light-absorbing chromophore. Upon activation, conformational changes associated with the FMN chromophore induce phototropin kinase activity^17^. Since their discovery, LOV domains have been found to be present in all forms of life (Archaea, Bacteria & Eukaryota), where they regulate a wide variety of functions including virulence and homeostasis^18^,^19^,^20^. Recently, Christie and co-workers enhanced the fluorescence and the photostability of the LOV domain from *Arabidopsis thaliana* phototropin 2 (phot2)^21^,^22^. The use of improved LOV (iLOV) or photostable iLOV (phiLOV) as fluorescent reporters has three main advantages over GFP as a reporter; phiLOV is smaller (~13 kDa vs ~25 kDa), fluorescence is stable over a greater pH range^23^, and fluorescence is not dependent on molecular oxygen^24^.

In addition, the discovery and mutation of LOV fluorescent reporters from diverse sources, each with different attributes^25^,^26^, are being increasingly exploited. For example, LOV domains from the freshwater algae *Chlamydomonas reinhardtii* (CreiLOV) have increased brightness over other LOV fluorophores^27^ whereas engineered LOV domains from *Arabidopsis thaliana* (phiLOV) have enhanced photobleaching-resistant properties^22^. As such LOV-based fluorescent proteins, together with other oxygen-independent reporters, including UnaG^28^ & mBFP^29^, are receiving increased attention as GFP alternatives and will also boost the bio-imaging toolkit in such systems.

In this paper, we show that phiLOV can be used as a tool to fluorescently label a number of obligate anaerobes including *Clostridium difficile, Clostridium sordellii* and *Clostridium acetobutylicum*. In addition, we reveal the potential of the phiLOV reporter to monitor the real time intracellular localisation and extracellular secretion of proteins within *C. difficile* through the generation of LOV translational reporter fusions. These data provide evidence of the wider utility of phiLOV technology, particularly in the study of pathogenesis within other anaerobic bacteria.

## Results

### Optimising the conditions for detecting phiLOV expression in *C. difficile*

The plasmid pRPF185-*phiLOV*, in which expression of a codon-optimised sequence of *phiLOV* was placed under the control of a tetracycline-inducible promoter, was introduced into *C. difficile* R20291 by conjugation (Supplemental Fig. 1a). To assess reporter activity, this strain was grown to mid-logarithmic phase and expression induced by the addition of 500 ng/ml anhydrotetracycline (ATc); a concentration which previously was shown to have limited impact on cell viability (This study &^30^). Expression of phiLOV by *C. difficile* was initially confirmed by Western blot analysis using antibodies to the iLOV protein (Supplemental Fig. 2).

**Figure 1:**
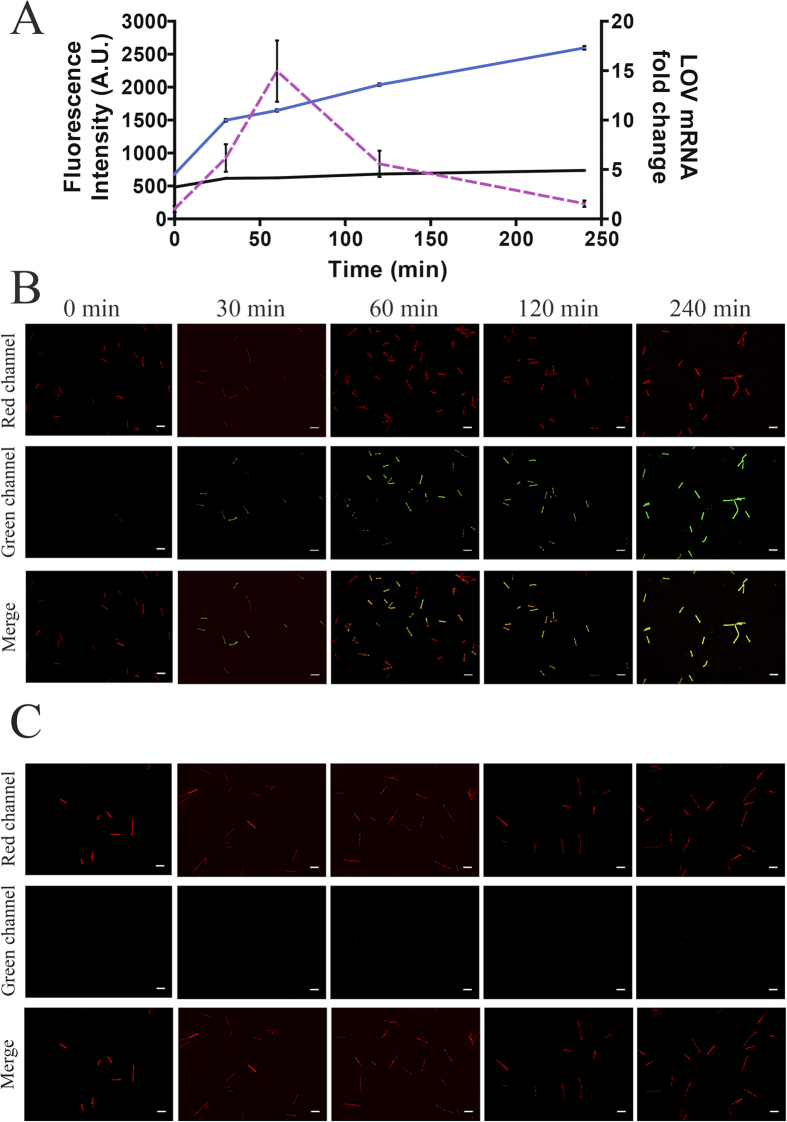
Characterisation of *phiLOV* expression in *Clostridium difficile* cells. (**A**) Relative fluorescence intensity (left *x*-axis) mediated by *phiLOV* (blue line) or *gusA* (black line) expressing cells. Samples were taken at 0, 30, 60, 120 & 240 min post induction with 500 ng/ml ATc, stained with FM4-64, fixed and viewed by fluorescence microscopy. Results shown are mean ± standard error of mean (SEM) of all bacterial cells from five de-convoluted fields of view and a minimum of two independent experiments. qRT-PCR analysis of *phiLOV* transcription (right *x*-axis) used RNA extracted at the same time points following induction from bacteria expressing *phiLOV* (dashed purple line). Expression represented as the fold change compared to pre-induction levels (0 min). Results shown are mean ± standard deviation (SD) of two independent experiments. Figures (**B,C**) are representative images of bacteria expressing either *phiLOV* (**B**) or *gusA* (**C**) following induction of expression for the durations shown in (**A**). LOV expressing strains generate green fluorescence whilst samples stained with FM4-64 (shown as red fluorescence) allow visualisation of the whole bacterial population. Images shown in (**B,C**) have undergone post image analysis (deconvolution and fluorescent contrast enhancement) to remove background autofluorescence. Scale bar in (**B**,**C**) 5 μm.

Following induction of expression through addition of ATc, these bacteria fluoresced green when excited with light of 470 nm. However, as *C. difficile* possess an intrinsic green autofluorescence when excited at this wavelength, we considered the relative increase in fluorescence of phiLOV over this background signal. This was achieved by analysis of fluorescence of *C. difficile* transformed with the plasmid pRPF185-*gusA*. In addition, the use of the membrane stain FM4-64, which has lipophilic binding activity, allowed visualisation of all bacteria in each field of view. Using the red fluorescence of this stain, it was possible to ensure that the equivalent numbers of LOV or GusA expressing bacteria were compared. Under these conditions, *C. difficile* containing the pRPF185-*phiLOV* plasmid, induced for 30 min with optimal concentrations of ATc, showed a relative 2.5-fold increase in fluorescence compared to those cells harbouring pRPF185-*gusA* alone (Fig. 1). In addition, the strength of the fluorescence signal increased over time reaching a relative 3.5-fold increase 240 min post induction. Figure 1b,c reflect representative images used in the analysis of relative fluorescence from *C. difficile* cultures expressing either *phiLOV* or *gusA*, respectively.

Whilst *phiLOV* expression in *C. difficile* could be clearly visualised, there was a concern that the detection systems used were not optimal. This is because the optimal excitation maxima (λ_max_) for phiLOV is 450 nm, however, for microscopic analysis the closest filter set available to us provided excitation at 470 nm. To determine the impact of changing the excitation wavelength on overall fluorescent signal, we induced strains expressing either *gusA* or *phiLOV* with 500 ng/ml ATc for 60 min. These samples were then analysed using a variable excitation/emission wavelength FluoroMax-3 spectrofluorometer. Using an excitation wavelength of 450 nm, *C. difficile* cells expressing *phiLOV* showed a 2.1-fold fluorescence increase compared to cells expressing *gusA* (*p* < 0.0001) whereas excitation at 470 nm showed a 1.7-fold fluorescence increase (*p* < 0.0001). These data show that whilst a significant difference was observed between *C. difficile* cells expressing either *gusA* or *phiLOV* when exciting at 470 nm, this difference could be further improved using a filter set that excites at 450 nm (Supplemental Fig. 3).

To further investigate the induction kinetics of *phiLOV* expression, the concentration of ATc was varied. This revealed that whilst an increase in concentration of ATc to 1 μg/ml did not significantly increase fluorescence activity, decreasing the concentration of ATc to 100 ng/ml, reduced the increase in fluorescence (1.9-fold higher fluorescence than control cells at 240 min). Thus under these conditions the optimal induction concentration for ATc was 500 ng/ml.

In parallel, measurement of *phiLOV* mRNA levels using qRT-PCR indicated a peak in transcript levels (approx. 15-fold higher relative to the control cells), 60 min post induction, after which *phiLOV* mRNA levels decreased (Fig. 1a dashed line). Interestingly this decrease was not associated with a reduction in fluorescence, providing further evidence to the enhanced stability of phiLOV fluorescence.

### Potential of phiLOV fluorescence in other *Clostridium* species

**Figure 2:**
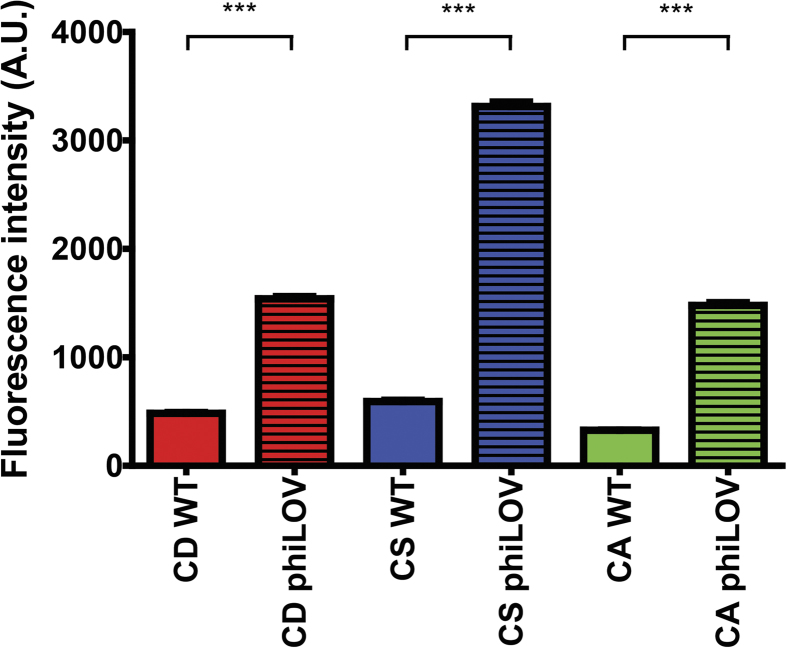
Expression of *phiLOV* in different *Clostridium* species. Comparison of the relative fluorescent intensities observed between plasmid-free *C. difficile* R20291 (CD WT), *C. sordellii* ATCC9714 (CS WT) & *C. acetobutylicum* ATCC824 (CA WT) and these strains expressing pRPF185-*phiLOV*. For this experiment, each *Clostridium* species were grown to *A*_600 nm_ of 0.5 and induced by the addition of 500 ng/ml ATc for 60 min. Results shown are mean ± standard error of mean (SEM) of all bacterial cells from five de-convoluted fields of view and a minimum of two independent experiments.

To confirm that phiLOV could be used as a fluorescent reporter in other *Clostridium* species, the phiLOV expression plasmid was introduced into *C. sordellii* and *C. acetobutylicum*. The strains were grown to *A*_600 nm_ 0.5 and induced with 500 ng/ml ATc for 60 min, fixed and examined by fluorescence microscopy. Induction of *phiLOV* expression resulted in a significant increase in fluorescence intensity of the population in each of the *Clostridium* species tested, compared to plasmid-free controls (*p* < 0.0001) (Fig. 2). Interestingly, expression of *phiLOV* in *C. difficile* saw the smallest fold-change in fluorescence intensity (3.2-fold increase) compared to *C. sordellii* or *C. acetobutylicum* (increase of 5.6- & 4.5-fold respectively), which probably reflects the lower levels of autofluorescence displayed by these strains (Fig. 2). This may suggest that this technology may have even greater potential application within these species.

### Use of phiLOV as a real-time protein reporter

**Figure 3:**
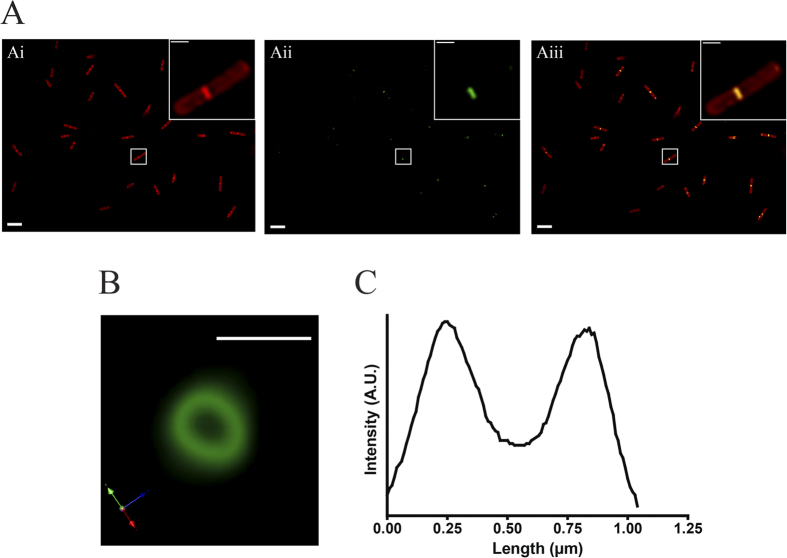
Monitoring FtsZ localisation using a FtsZ-phiLOV protein reporter. (**A**) Localisation of fluorescence to cell midpoints in *C. difficile* expressing the FtsZ-phiLOV and grown under standard induction conditions. (Ai) the bacterial population stained with FM4-64; (Aii) green fluorescence as a consequence of LOV expression and (Aiii) merged images. Images underwent post image analysis (deconvolution and fluorescent contrast enhancement) to remove background autofluorescence. Scale bars in 5 μm and, in insert 1 μm. (**B**) Representation of the FtsZ-phiLOV ring in 3D using compiled data from z-stack images. Z slices were taken at 0.2 μm intervals through the bacterial cell and 3-dimensional analysis completed using max intensity 3D rendering in Volocity. Scale bar, 1 μm. (**C**) Fluorescent intensity profile through the FtsZ-phiLOV ring shown in (**B**). This reflects the peak fluorescence intensity plotted against distance.

To determine whether phiLOV could be used as a real-time reporter for monitoring protein localization and dynamics within a bacterial cell, we genetically fused this protein to the C-terminus of the cytokinetic protein FtsZ of *C. difficile* (Supplemental Fig. 1b). FtsZ is involved in the formation of a cytokinetic ring (or Z-ring) at the site of cell division^31^ and has successfully been tagged with GFP in other bacterial species^32^. Western blot analysis using anti-iLOV antibody confirmed that *C. difficile* successfully expressed the FtsZ-phiLOV fusion protein (Supplemental Fig. 2). To determine whether the protein retained functionality, bacteria were grown to mid-logarithmic phase (when cells are most actively dividing) and induced with ATc for 30 minutes. Cell membrane was additionally visualised by co-staining these cells with the lipophilic dye FM4-64. Fluorescent imaging of these organisms revealed a fluorescent signal detected at the cell mid-point in ~77% of bacterial cells undergoing cell division (Fig. 3a). A 3-dimensional recreation of the FtsZ-phiLOV fluorescent signal from z stack images further resolved the mid-cell fluorescent signal into a ring structure (Fig. 3b). Measuring the signal intensity across the diameter of this ring confirmed this structure to be ~1 μm in diameter (Fig. 3c).

**Figure 4:**
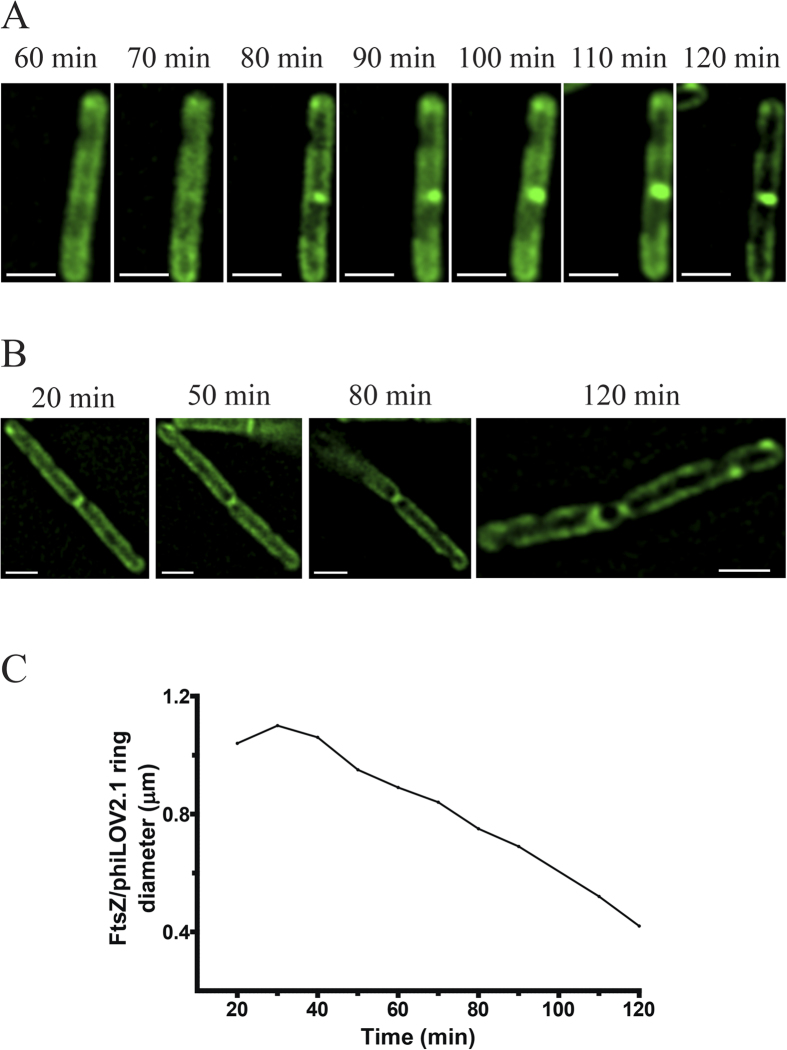
Real time Z ring assembly and constriction in *C. difficile* using fluorescent microscopy. (**A**) FtsZ-phiLOV incorporation during Z-ring assembly in real time using images of the same cell captured every 10 min. (**B**) Constriction of the Z ring within a bacterial cell during cell division over 120 min. Organisms shown in the images were induced with 500 ng/ml ATc and immobilised on an agarose pad under a sealed cover slip for time-lapse observation. Images have undergone post image analysis (deconvolution and fluorescent contrast enhancement). Scale bar in (**A,B**) 2 μm. (**C**) Changes in Z ring diameter over time. These ring diameter measurements were taken from images shown in (**B**).

One of the main advantages of phiLOV over other reporter systems is their potential as real time reporters for protein dynamics. To this end, we induced *C. difficile* harbouring pRPF185-*FtsZ-phiLOV* and immediately immobile cells on agarose pads, which were covered and sealed within the anaerobic chamber. Using this technique, *C. difficile* cells can be transferred without impact on viability to aerobic environments for imaging. In this way we were able to observe the incorporation of FtsZ-phiLOV monomers during assembly of the Z ring in dividing cells and constriction of the Z ring (Supplemental Fig. 4). Time-lapse imaging of a bacterial cell showed accumulation of fluorescent signal at the cell midpoint approximately 70 min post induction. This started at one point on the circumference of the bacterial cell and took ~40 min to increase the length of the FtsZ-phiLOV arc to span the cell (Fig. 4a). Once a complete Z ring had formed, constriction of this ring preceded the narrowing of the cell diameter as membrane biogenesis occurred at the site of division, a process that took ~80 min to complete (Fig. 4b). Constriction of the Z ring, certainly in the later stages of constriction, proceeded at a linear rate (Fig. 4c).

### Use of phiLOV protein fusions to track exported proteins

**Figure 5:**
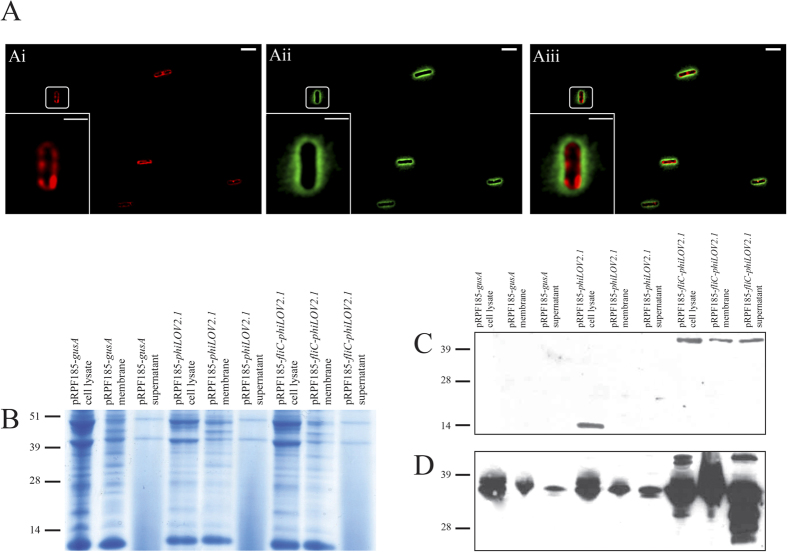
Secretion of FliC-phiLOV fusion proteins in *C. difficile*. (**A**) Localisation of fluorescence in *C. difficile* expressing the *fliC-phiLOV* fusion protein. Bacteria were grown to *A*_600_ _nm_ of 0.5, induced with 500 ng/ml ATc, co-stained with FM4-64 and immobilised on agarose pads as described. (Ai) bacterial population stained with FM4-64; (Aii) green fluorescence as a consequence of LOV expression and (Aiii) the merged images. Extended focus displays a brightest point merge of all planes of representative images of *C. difficile* expressing *fliC-phiLOV* fusion protein. (Supplemental Fig. 5 for z slices). Images shown have undergone post image analysis (deconvolution and fluorescent contrast enhancement) to remove background autofluorescence. Scale bar, 5 μm and, in insert, 2 μm. (**B**) SDS-PAGE of cell lysate, membrane extracts and culture supernatants from *C. difficile* expressing either *gusA*, *phiLOV* or *fliC-phiLOV*. Western blot analysis of protein expression using either anti-iLOV antibody (**C**) or anti-FliC antibody (**D**) as the probes. Predicted protein sizes of phiLOV, FliC and FliC-phiLOV are 13, 30.9 and 43.7 kDa, respectively, however FliC is known to be post-translationally modified^35^ so size will vary. Molecular weights expressed in kDa.

As previously noted, phiLOV is approximately half the size of the GFP-family of fluorescent proteins and as such maybe sufficiently small to be used in protein-membrane translocation studies. To establish whether phiLOV could be used in this context in *C. difficile*, the sequence was cloned into a central site within the major flagellin protein FliC (Supplemental Fig. 1c). To limit complications of native FliC expression, this construct was transferred into strain *C. difficile* 630 in which *fli*C had been deleted (630 Δ*fliC*) as well as wild-type cells (630). Induced cultures were stained with FM4-64 prior to fixation and examination by fluorescence microscopy. Z stack imaging of these cells showed little to no intracellular fluorescence (Supplemental Fig. 5), but instead an accumulation of fluorescence that appeared to be external to the cytoplasmic membrane of these cells (Fig. 5a). To determine whether the protein was exported into the media, western blot analysis was performed on samples recovered from the cytosolic and membrane fractions of the bacterial cells or from filtered extracts of the culture supernatant (Fig. 5b). Using antibodies to both iLOV and FliC respectively, immunoreactive bands of approximately 44 kDa were identified in samples generated from the cytosolic, membrane and culture supernatant of *C. difficile* cells expressing *fliC-phiLOV*. In contrast, an immunoreactive band of 13 kDa was only observed in the cytosolic extract of cells expressing *phiLOV* alone (Fig. 5c,d). These data help to confirm the observation that phiLOV is able to translocate the membrane when fused to the FliC momomer. In addition, the FliC antibody recognised a protein of approximately 35 kDa, the approximate size of a post-translationally modified FliC monomer. In addition, several breakdown products were observed in the strain expressing *fliC-phiLOV*. This probably reflects degradation of poorly folded proteins following the relatively unregulated overexpression of the protein from the inducible tetracycline promoter.

While expression of *fliC-phiLOV* in *C. difficile* appears to result in export from the cells, expression did not restore flagella mediated motility or result in detectable flagella filaments on the cell surface (data not shown). This most likely reflects an inability to correctly fold FliC as a consequence of phiLOV insertion. This could prevent the appropriate polymerization of the FliC monomers into the flagellar structure resulting in their subsequent release and recovery in the filtered media from the culture.

## Discussion

In this study, we report the first use of a LOV domain as a fluorescent reporter in the anaerobic bacterium *C. difficile*. Whilst its relative small size (~13 kDa) makes it attractive as a reporter, its green fluorescence had been thought to limit its potential application in naturally autofluorescent bacteria, such as *C. difficile*. Further, unlike the GFP/RFP-family of fluorescent proteins, mutational analysis of LOV reporters has largely failed to shift the excitation/emission spectra, with only small spectral blue shifts in fluorescence emission (~10 nm) reported^25^,^26^. Also LOV-based FPs engineered thus far exhibit fluorescence quantum yields (Q_F_) that are significantly lower (~0.2–0.5) to that of GFP (0.6)^23^, which could impact their utility for certain reporter applications. Despite these issues, we report that a differential level of fluorescence due to phiLOV expression is quantifiable with fluorescence appearing stable over time. In addition, the use of phiLOV as a reporter in this context does not appear limited to *C. difficile* with differential expression also observed in other *Clostridium* species, including the commercially important *C. acetobutylicum* in which lower levels of autofluorescence are observed.

Further, we have demonstrated that phiLOV can be used as a reporter of protein expression within *C. difficile*, using two model proteins FtsZ and FliC. This is the first time that protein expression has been visualised in real time by fluorescence in *C. difficile*, with previous used GPF-based reporters being restricted by the requirement for oxygen. Using time-lapse fluorescent microscopy, we show the formation of FtsZ-phiLOV z ring and additionally the constriction of the Z ring in real time, taking approximately 130 min to complete the whole process. The genetic fusion of *phiLOV* to *ftsZ* appeared to have no detrimental effects on FtsZ function with FtsZ-phiLOV able to polymerise into a ring structure, anchor to the cell membrane, hydrolyse GTP & interact with other proteins in the divisome. Whilst study of this phenomenon in real-time was technically difficult to perform, requiring maintenance of anaerobiosis, the images highlight the integral robust stability of the phiLOV fluorophore. This stability^25^, is an important feature when considering time lapse measurements.

Resolution of this fluorescence using z-stack analysis, deconvolution and 3D rendering confirmed the formation of a ring-like structure and further underlines the potential of phiLOV as a reporter for protein localisation. Observation of several fields of non-synchronised dividing bacteria also revealed the presence of incomplete rings in some bacteria. This is unsurprising given that these bacteria contain both plasmid-encoded *FtsZ-phiLOV* and the intact chromosomal *ftsZ* locus. Consequently, these incomplete rings may reflect structures composed of both labelled and unlabelled FtsZ.

The small size of LOV proteins, when compared to GFP, has the potential to be particularly useful when creating translational fusions especially for the study of proteins that are secreted from the cytoplasm. To evaluate the potential of phiLOV in this context we generated a *fliC-phiLOV* construct, in which *phiLOV* was inserted at nucleotide 483 of the *C. difficile fliC* sequence. In other bacterial species, the proposed protein folding and monomer polymerisation of FliC molecules is thought to include the N- & C-terminal sequences of the FliC protein^33^ with the central portion of the monomer exposed on the flagella surface. Visualisation using Z stack images (Supplementary Fig. 5) taken through the entire bacteria revealed fluorescence on the periphery of those bacteria expressing the fliC/LOV fusion, which suggested that the fusion of phiLOV did not hinder secretion of FliC subunits. This observation was surprising but interestingly also observed at much lower levels in the control strains. Disappointingly, expression and secretion of this this fusion did not result in any detectable flagella filaments or restore motility to the bacterium. These data suggest that the presence of the tag is limiting the opportunity for effective subunit polymerisation rather than secretion. This problem may reflect the chosen location for insertion of the tag. Indeed, whilst the fusion protein was detected in the cytoplasm, membrane fractions and filtered spent growth medium, several related breakdown products were also detected using the specific FliC antibodies. This would suggest that the rate of expression might influence the protein folding and the potential of these proteins to polymerise into definitive flagellar structures. Thus this work shows that although phiLOV can be used to label proteins that are translocated across the membrane, the location of the tag and the regulation of protein expression may influence the incorporation of such fusion proteins into macromolecular structures.

Whilst the examples given here highlight the opportunities offered by the use of phiLOV, the natural autofluoresence of *C. difficile* is likely to restrict its use for study of low abundance proteins. Whilst this issue cannot be avoided, we have attempted to quantify the level of *phiLOV* expression needed to detect a significant change in *C. difficile* fluorescence; in our system, we calculated that a 2.68 fold-increase in *phiLOV* mRNA levels correlated with a detectable increase in fluorescence. Although based on several assumptions, the calculated expression fold-increase can give an indication of the level of target expression needed before phiLOV expression is observed only further experimentation will allow us to establish the scope of its use. In parallel, the application of fluorophores, which are excited by wavelengths outside the blue/green spectrum, such as SNAP tags (based on O^6^-alkylguanine-DNA alkyltransferase^9^), CFP (a GFP-family protein^8^) or mCherry (a Red Fluorescent Protein (RFP)-family protein^7^) are important developments. However, the use of these fluorescent systems in anaerobic environments requires either a fixation step before fluorescence detection or exposure to molecular oxygen, limiting their use in the study of protein dynamics in real time. Using the time-lapse experiments described, we show that LOV domains can bridge this limitation, allowing researchers the opportunity to study protein dynamics in anaerobic systems in real-time. As such, we propose that at least for some situations, a combination of both systems is appealing, potentially allowing the amount and location of specific proteins (using reporters systems described above) at specific time points determined by linkage of phiLOV to proteins, for example associated with cell cycle.

In conclusion, this study highlights the use of the phiLOV domain as a fluorescent reporter in the anaerobic genus *Clostridium*. Furthermore, translational fusions that include these domains can be used to study protein localisation and dynamics in real-time and offer opportunities to increase our understanding of protein dynamics in response to environmental stimuli. In addition, as we advance our capacity to identify and culture a much wider number of strictly anaerobic bacteria, the availability of fluorescent technologies that can be readily applied under hypoxic conditions is attractive. As a result, the potential to apply phiLOV technology is wide ranging and may play a significant role in enhancing our understanding of gene expression and regulation in many important but poorly understood anaerobic organisms.

## Materials and Methods

### Bacterial strains and growth

Bacterial strains used in this study are listed in Supplemental Table 1. *Escherichia coli* TOP10 (Invitrogen, U.K.), used as a cloning host and *E. coli* CA434^34^ as a conjugal donor were grown aerobically on Luria Bertani medium supplemented with ampicillin (100 μg/ml) or chloramphenicol (15 μg/ml) when required. *C. difficile* strains R20291 & 630 Δ*fliC*^35^ were kind gifts from Prof. B. Wren and Dr Alex Faulds-Pain (London School of Hygiene and Tropical Medicine, London, U.K.). *C. acetobutylicum* strain ATCC824 was purchased from the American Type Culture Collection (ATCC). *C. difficile* strains were routinely grown on CCEY agar plates supplemented with cefoxitin-cycloserine, egg emulsion (Lab M Ltd,), and if required 15 μg/ml thiamphenicol, in an anaerobic workstation (Don Whitley Scientific Ltd, U.K.) at 37 °C. Brain-heart infusion (BHI) broth or Tryptone Yeast (TY) broth (supplemented with 15 μg/ml thiamphenicol) were routinely used to grow *C. difficile* strains during fluorescent experiments. *C. sordelli* was cultured in TY broth and *C. acetobutylicum* strain ATCC824 grown using Reinforced Clostridium Media (RCM, Lab M Ltd), both supplemented, when required, with 15 μg/ml thiamphenicol. Plasmids were introduced into *C. difficile* and *C. sordellii* by conjugation from *E. coli* CA434 as previously described^34^. *C. acetobutylicum* was transformed with pRPF185-*phiLOV2.1* by Dr Liz Jenkinson (Green biologics Ltd, U.K.).

### Cloning and plasmid construction

#### *Construction of* phiLOV2.1 *construct*

The *phiLOV2.1* gene^21^ was codon optimized for expression in *C. difficile* (GenScript USA Inc.), and introduced into vector pUC57 (Supplemental Table 1). Primers 69F & R (Supplemental Table 2) were the used to amplify *phiLOV2.1*, adding *Sac*1 & *Bam*H1 restriction enzyme sites to allow ligation of the sequence downstream of the *tet* promoter, within the pRPF185 vector^30^. The resultant plasmid, pRPF185-*phiLOV2.1*, was then transformed into *E. coli* TOP10 cells (Invitrogen Ltd). To confirm the sequence of the cloned fragment, primers 73F & 72R were used to amplify the region from the plasmid and the resultant product, purified using the Qiagen PCR purification kit and then subjected to sequence analysis.

#### *Construction of C-terminal* FtsZ-phiLOV2.1 *fusion*

phiLOV2.1 was genetically linked to the C-terminus of the cytokinesis protein FtsZ using primers 97F/R & 98F/R (Supplemental Table 2). These primers amplified the *ftsZ* and *phiLOV2.1* sequences respectively, adding *Nhe*1 restriction enzyme sites to the 3′ and 5′ ends of the *fts*Z and *phiLOV*2.1 sequences respectively. The PCR fragments were then ligated using the complementary *Nhe*I sites and the resultant *ftsZ-phiLOV2.1* fragment further amplified using primers 99F & R before it was cloned into pRPF185 and sequenced as described.

#### *Construction of* fliC-phiLOV2.1 *fusion*

*phiLOV2.1* was linked to the major structural flagella protein encoded by *fliC* by inserting the *phiLOV2.1* sequence between nucleotides 483–484 of *fliC*. Primers 133F & R and 135F & R were used to independently amplify the first 483 nucleotides and last 390 nucleotides of the *C. difficile* 630 *fli*C gene encoded by *C. difficile* 630. These primers were additionally designed to incorporate complementary sequences to those found in *phiLOV2.1* sequences. In parallel, primers 135F &134R (Supplemental Table 2) were designed to amplify *phiLOV2.1* whilst additionally removing the stop codon from this protein and add overlapping complementary *fliC* sequences. The three fragments were then amplified by PCR, purified and assembled using the Gibson Assembly® Mastermix kit (New England Biolabs Inc.). The ligated *fliC-phiLOV2.1* construct underwent a further round of PCR amplification using primers 139F & 140R to allow the addition of homologous overlapping sequences allowing subsequent ligation of the sequence into the expression vector pRPF185.

### Fluorescence microscopy

To allow the entire bacterial population to be visualised by fluorescence, the membrane dye FM4-64 (Invitrogen) was added to samples taken from each culture at a final concentration of 5 μg/ml (10 min, 4 °C). Stained cells were washed by centrifugation in distilled water and then fixed in 10% formalin (Sigma) for 15 min in the dark. The bacteria were then washed in sterile distilled water before being air-dried onto glass slides. Prepared slides were mounted in Dako Fluorescent Mounting Medium (Dako Ltd) before addition of a glass cover slip. For time-lapse imaging, appropriate *C. difficile* strains were treated with 500 ng/ml ATc before being immobilised onto thin 1% agarose pads, which were covered with a glass cover slip that was sealed with Dako Fluorescent Mounting Medium. For these experiments, mounting and sealing of the slides was performed within the anaerobic cabinet to ensure viability of the bacteria. Bacterial cells were examined using an Axio-Zeiss Imager M1 light microscope (Carl Zeiss Microscopy GmbH) and images captured using a Hamamatsu ORCA-ER digital camera with motorized excitation and emission filter wheels and Zen 2012 (blue edition) acquisition software (Carl Zeiss Microscopy GmbH). Slides were exposed for a maximum of 500 ms (unless otherwise stated) using an excitation beam of 470 nm, emission was detected at 520 nm to detect phiLOV2.1 fluorescence and an excitation 540 nm, emission 725 nm to detect FM4-64 fluorescence. Z-stack experiments were performed at distances of 0.2 μm. Deconvolution of raw data was performed using a point-spread function with 30 rounds of iteration. Under the excitation/emission spectra used for phiLOV2.1 detection, *C. difficile* naturally fluoresce green. To determine the impact of this background fluorescence on the data, the mean background control fluorescence of same strain of *C. difficile* transformed with the control plasmid, pRPF185-*gusA,* was calculated from a minimum of 5 fields of view in three independently performed experiments. The mean fluorescence intensity of every bacterial cell imaged was determined using Volocity 3D image analysis software version 5.5 (PerkinElmer Inc., U.S.). Results represent the mean population fluorescence intensity ± standard error of mean from five fields of view. 3D image rendering for the FtsZ-phiLOV2.1 Z ring structure was performed using Volocity 3D image analysis software.

### Statistical analysis

All statistical analyses were performed using the GraphPad Instat 3.10 (GraphPad Instat Software). A Students T-test was used to determine significant differences in fluorescence emissions between the different strains. *P* values ≤ 0.05 were considered significant.

### Spectroscopic analysis

Fluorescence excitation and emission spectra were recorded using a FluoroMax-3 spectrofluorometer (Horiba Scientific) and a scan speed of 100 nm/min as described previously^21^.

### Cell lysis, fractionation and protein analysis

To determine the expression and location of each recombinant protein, each culture was grown in BHI to an *A*_600 nm_ of approximately 0.6 O.D. units before induction of protein expression with ATc for 4 h. The cultures were then harvested by centrifugation (500 g, 10 min). The supernatant was removed and immediately filtered using 0.2 nm syringe filter. Proteins within the culture supernatant were concentrated using the TCA/acetone precipitation method as previously described^36^. The remaining cell pellet was immediately frozen and cytosolic and membrane fractions separated as described^30^. In brief, samples were prepared to ensure equivalent loading, which was achieved by re-suspending the pellets in PBS containing lysozyme (1.4 mg/ml) and DNAse 1 (120 mg/ml) to provide an OD_600_ = 20. Membranes and cytoplasmic fractions were then separated by high-speed centrifugation (25,000 g). Whilst the supernatant was removed immediately and mixed with 2× SDS sample buffer, the resultant membrane fractions were then washed twice in PBS before being solubilised in 1% SDS to an equivalent volume of OD_600_ = 20. These samples were finally mixed with an equal volume of 2× SDS sample buffer. SDS-PAGE and Western blotting analysis were performed using standard methods with protein samples separated using 12% SDS-PAGE gels (Novex, Life technologies). After transfer to Hybond-C nitrocellulose membrane (Amersham Biosciences), blots were either probed using rabbit anti-iLOV antibody (1:1000)^21^ followed by HRP-linked secondary goat anti-rabbit antibody (1:3000; Sigma) or by chicken anti FliC (a kind gift from Glen Armstrong) (1:5000) detected by goat anti chicken Y-chain HRP conjugate (1:15000). HRP activity was subsequently detected using LumiGLO® (New England Biolabs Inc.).

### RNA extraction and purification

qRT-PCR was performed on mRNA samples isolated from mid-log cultures induced with ATc as described previously^37^. In brief, mRNA was extracted using the FastRNA Pro Blue Kit (MP Biosciences) from bacterial cultures, maintained under anaerobic conditions. Initially, anaerobic cultures were treated with 10 ml RNA*later*® (Ambion) for 5 min before the bacteria were collected by centrifugation (5000 × g; 10 min; 4 °C). The bacterial pellets were then re-suspended in 1 ml RNA Pro solution and transferred to matrix tubes in which bacteria were lysed using a FastPrep-24 instrument (MP Biosciences); (2 × 45 s at 6.0 ms^−1^). To minimise degradation, samples were cooled on ice for 5 min between cycles. The samples were subject to centrifugation (12000 × g; 10 min; 4 °C), and nucleic acid recovered from the supernatant, which was placed into an RNAse free tube to which 300 μl chloroform was added. The resultant mixture was vortexed and then centrifuged (12000 × g; 15 min; 4 °C) and the aqueous phase was separated and transferred to a fresh tube containing 500 μl of cold absolute ethanol. This was inverted and incubated −20 °C for 30 min. After centrifugation (12000 × g; 15 min; 4 °C) the pellet was washed with 500 μl of cold 75% ethanol and air dried for 5 min at room temperature. DNase treatment was carried out using the rigorous Turbo DNase kit (Ambion) according to manufacturer’s instructions. The RNA was further cleaned using the RNeasy Mini kit (Qiagen) and RNA purity and quantity assessed by nanodrop UV spectroscopy.

### Analysis of relative levels of mRNA expression

For semi quantitative qRT-PCR, first strand cDNA was synthesized from total RNA using random hexamers as primers and the SuperScript^TM^ III Reverse Transcriptase (Invitrogen) according to manufacturer’s instructions. qRT-PCR was performed in triplicate in a 20 μl reaction volume containing 200 ng of cDNA, 10 μl Fast SYBR Green Master Mix (5 Prime GmbH) and 500 nM gene specific primers (Supplemental Table 2) in an Illumina Eco^TM^ real-time PCR instrument (Illumina). In each sample, the quantity of LOV specific cDNAs were normalised to the quantity of cDNAs of the *rpo*A gene. The relative transcript changes were calculated using the 2^−ΔΔCt^ method^38^.

## Additional Information

**How to cite this article**: Buckley, A. M. *et al.* Lighting Up *Clostridium Difficile*: Reporting Gene Expression Using Fluorescent Lov Domains. *Sci. Rep.* **6**, 23463; doi: 10.1038/srep23463 (2016).

## Supplementary Material

Supplementary Information
