# Supplementary material for: Lighting Up Clostridium Difficile: Reporting Gene Expression Using Fluorescent Lov Domains
Source: Sci Rep. 2016 Mar 21;6:23463. doi: 10.1038/srep23463 (PMC4800718; doi:10.1038/srep23463)
Supplement: Supplementary Information [file srep23463-s1.pdf]

LIGHTING UP *CLOSTRIDIUM DIFFICILE*: REPORTING GENE EXPRESSION USING FLUORESCENT LOV DOMAINS

Anthony M. Buckley, Caitlin Jukes, Denise Candlish, June J. Irvine, Janice Spencer, Robert P. Fagan, Andrew J. Roe, John M. Christie, Neil F. Fairweather & Gillian R. Douce

Supplemental Table 1 Douce.

Strain/Plasmid	Characteristics	Reference/Source
**Strains**		
E. coli		
TOP10	F¯ *mcrA* Δ(*mrr-hsdRMS-mcrBC*) φ80*lacZ*ΔM15 Δ*lacX74 nupG recA1 araD139* Δ(*ara-leu*)*7697 galE15 galK16 rpsL*(Str^r) *endA1* λ¯	Invitrogen
CA434	*E. coli* HB101 [F¯ *mcrB mrr hsdS20*($r_B^- m_B^-$) *recA13 leuB6 ara-14 proA2 lacY1 galK xyl-5 mtl-1 rpsL20*(Sm^r) *glnV44* λ¯]	Ref:33
C. difficile		
R20291	Wild-type; PCR ribotype 027 (Stoke Mandeville, U.K., epidemic strain)	B. Wren, LSHTM, London, U.K.
630	Wild-type; PCR ribotype 012 (Zurich, U.K.)	B. Wren, LSHTM, London, U.K.
630 Δ*fliC*	630 *fliC::[ClosTron, ermB]*	Ref:34
R20291 pRPF185-*gusA*	R20291 harbouring pRPF185-*gusA*	This study
R20291 pRPF185-*phiLOV*	R20291 harbouring pRPF185-*phiLOV2.1*	This study
R20291 pRPF185-*ftsZ-phiLOV*	R20291 harbouring pRPF185-*ftsZ-phiLOV2.1*	This study
630 pRPF185-*fliC-phiLOV*	630 harbouring pRPF185-*fliC-phiLOV2.1*	This study
630 Δ*fliC* pRPF185-*fliC-phiLOV*	630 Δ*fliC* harbouring pRPF185-*fliC-phiLOV2.1*	This study
C. sordellii		
ATCC9714	Type strain; $TcsL^+$ $TcsH^-$	N. Fairweather, UCL, London, U.K.
ATCC9714 pRPF185-*phiLOV*	ATCC9714 harbouring pRPF185-*phiLOV2.1*	This study
C. acetobutylicum		
ATCC824	Type strain	ATCC
ATCC824 pRPF185-*phiLOV*	ATCC824 harbouring pRPF185-*phiLOV2.1*	L. Jenkinson, Green Biologics Ltd, U.K.
**Plasmids**		
pRPF185-*gusA*	Anhydrotetracycline-inducible (P_{tet}) vector for *C. difficile* expressing *gusA*	Ref:30
pUC57-*phiLOV*	High copy number vector containing the codon optimized *phiLOV2.1*	Ref:21 & GenScript
pRPF185-*phiLOV*	*gusA* gene in pRPF185 replaced with codon optimized *phiLOV2.1*	This study

pRPF185-*ftsZ-phiLOV*	*gusA* gene in pRPF185 replaced with *C. difficile* R20291 *ftsZ* with 3' *phiLOV2.1* modification	This study
pRPF185-*fliC-phiLOV*	*gusA* gene in pRPF185 replaced with *C. difficile* 630 *filC* with *phiLOV2.1* modification	This study

Supplemental Table 2 Douce.

Primer Name	Use	Sequence (5’-3’)	Reference/ Source
69F	Amplification of *phiLOV2.1* and addition of RE sites	GCGAGCTCAAATTTGAATTTTTTAGGGGGAAAATACCATGATTGAAAAAGTTTTGTTATTAC	This study
69R		CATGGATCCTTATTAAACATGATCTG	
73F	Insert confirmation and sequencing primers for plasmid pRPF185	CTGGACTTCATGAAAAACTAAAAAAAAATATTG	30
72R		CACCGACGAGCAAGGCAAGACCG	
97F	Amplification of *ftsZ* and addition of RE site for C-terminal tag	ATGATGCTAAACTTTGACGT	This study
97R		GCATGCTAGCAGGTCCTCTTCTTCTTCTTAGGAATGTAGGTATCTCC	
98F	Amplification of *phiLOV2.1* and addition of RE site for C-terminal tag	TGCGCTAGCCCAGGTATGATTGAAAAAGTTTTGTTATTACTG	This study
98R		TTAAACATGATCTGAACCAAC	
99F	Amplification of C-terminal tag *ftsZ-phiLOV2.1* insert and addition of RE sites	GTCGAGCTCAAATTTGAATTTTTTAGGGGGAAAATACCATGCTAAACTTTGACGTAGAATTAGAAGAATGTGC	This study
99R		CAATGGATCCGGAATTAAACATGATCTGAACC	
133F	Amplification of first 483 bp of *fliC* and addition of 5’ *fliC* RBS & 3’ *phiLOV2.1* sequence for Gibson assembly®	TACTGAGCTCACAAGGATGTCAACTATAC	This study
133R		CAGTAATAACAAAACTTTTTTCAATCATATTATTACTTGTACCTGAAACATTAGTTCC	
134F	Amplification of *phiLOV2.1* and addition of 5’ & 3’ *fliC* sequences for Gibson assembly®	GGAACTAATGTTTCAGGTACAAGTAATAATATGATTGAAAAAGTTTTGTTATTACTG	This study
134R		GCAGTATTTACTAATTGAATTTTTATCTCATTGTTAACATGATCTGAACCAACTAATTGAACTCC	
135F	Amplification of second 390 bp of *fliC* and addition of 5’ *phiLOV2.1* sequence for Gibson assembly®	GGAGTTCAATTAGTTGGTTCAGATCATGTTAACAATGAGATAAAAATTCAATTAGTAAATACTGC	This study
135R		GTTAGGATCCTTGTTACTATCCTAATAATTGTAAAAC	
139F	Amplification of *fliC/phiLOV2.1* introducing Gibson assembly® sequences for plasmid ligation	CGTAGCGTTAACAGATCTGAGCTCACAAGGATGTCAAC	This study
140R		TAAAACTTATAGGATCCTTGTTACTATCCTAATAATTG	

RE, restriction enzyme; RBS, ribosome binding sequence

SUPPLEMENTAL FIGURE LEGENDS

Supplemental figure 1. Plasmid maps constructed in this study. Inducible plasmid pRPF185[30] was used as a backbone for plasmid construction. **A.** *C. difficile* codon optimised *phiLOV2.1* was cloned using *Sac*1 and *Bam*HI restriction enzymes and insertion confirmed by sequencing. Reporter fusions of C-terminal *ftsZ-phiLOV2.1* (**B**) and *fliC-phiLOV2.1* (**C**). *In silico* plasmid maps were constructed using CLC Genomics Workbench version 7 (CLC bio, U.S.)

Supplemental figure 2. phiLOV2.1 detection by Western blot analysis. *C. difficile* expressing either *gusA*, *phiLOV2.1* or *ftsZ-phiLOV2.1* were induced with 500 ng/ml ATc for 30 min prior to the use of anti-iLOV specific antibody to confirm the production of phiLOV2.1 and fusion proteins. Molecular weights expressed in kDa.

Supplemental figure 3. Characterisation of excitation and emission spectra of *C. difficile* pRPF185-*phiLOV2.1*. A. Comparison of *C. difficile* GusA and phiLOV2.1 emission fluorescence when excited at either 450 nm (blue bars) or 470 nm (red bars). Emission fluorescence detected at 520 nm. **B.** Relative fluorescence intensity of *C. difficile* pRPF185-*gusA* (dashed lines) or pRPF185-*phiLOV2.1* (whole lines) over time, as measured using a spectrofluorometer. Bacterial cells were exposed to 500 ng/ml ATc for 0 min (black line), 30 min (orange line), 60 min (yellow line), 120 min (red line) and 240 min (blue line). Bacterial cells were exposed to an excitation wavelength of 470 nm and emission spectra measured using a scan speed of 100 nm/min.

Supplemental figure 4. Sequential time-lapse fluorescence microscopy images after induction of *ftsZ-phiLOV2.1* expression. White arrowheads denotes the bacterial cell used for FtsZ-phiLOV2.1 ring assembly measurements (starting at 60 min post induction) whilst blue arrows denotes the bacterial cell used for FtsZ-phiLOV2.1 ring constriction

measurements. Bacterial cells were immobilised using 1 % agarose pads after induction with 500 ng/ml ATc. Conditions were kept anaerobic using a glass cover slip and mounted using Dako fluorescent mounting medium. Scale bar is 5 µm.

Supplemental figure 5. *C. difficile* extracellular fluorescence after secretion of FliC-phiLOV2.1. Sequential z stack images of *C. difficile* pRPF185-*fliC*/*phiLOV2.1* cells. Cells were induced with 500 ng/ml ATc, co-stained with FM4-64 and immobilised on agarose pads before fluorescent microscopy. Z stack images were taken at 0.28 µm intervals. Scale bar is 5 µm.

A

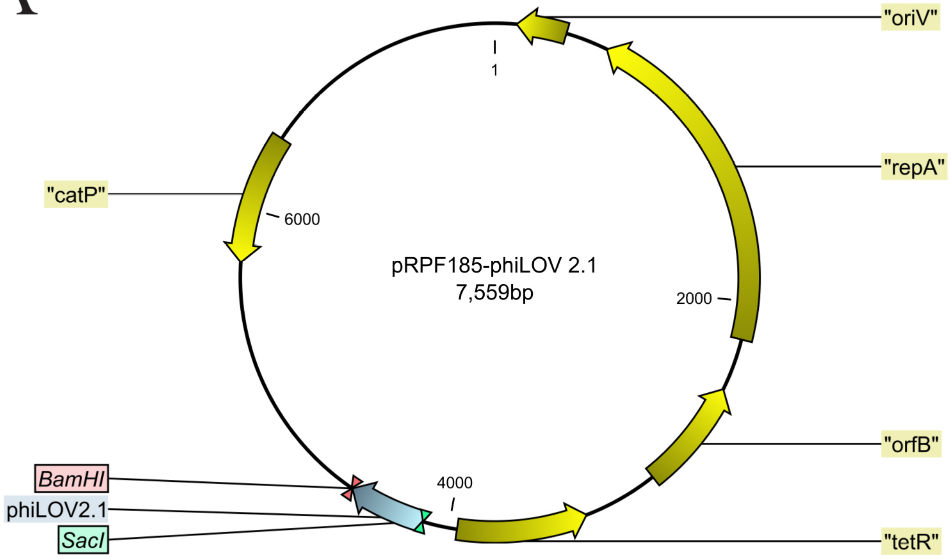

"oriV"
1
"repA"
"catP"
6000
pRPF185-phiLOV 2.1
7,559bp
2000
"orfB"
BamHI
4000
phiLOV2.1
SacI
"tetR"

B

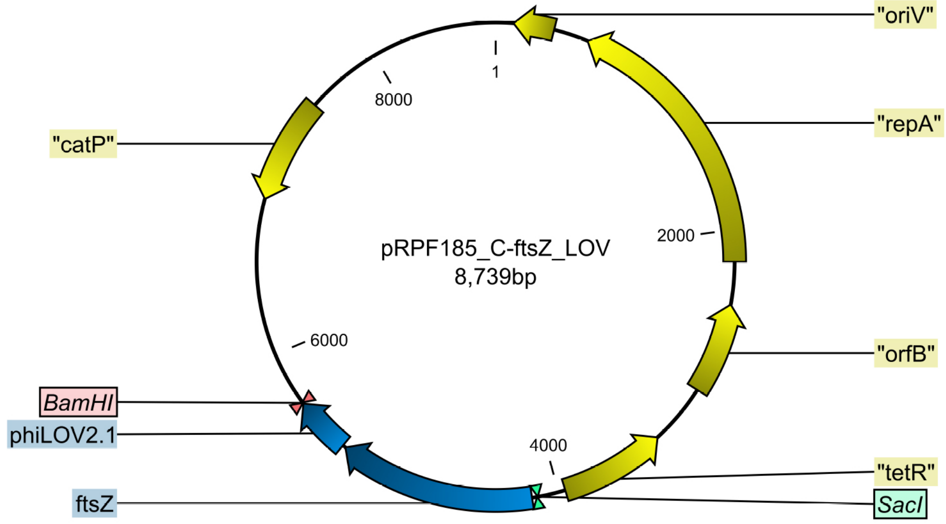

"oriV"
1
8000
"repA"
"catP"
pRPF185_C-ftsZ_LOV
8,739bp
2000
6000
"orfB"
BamHI
phiLOV2.1
4000
"tetR"
ftsZ
SacI

C

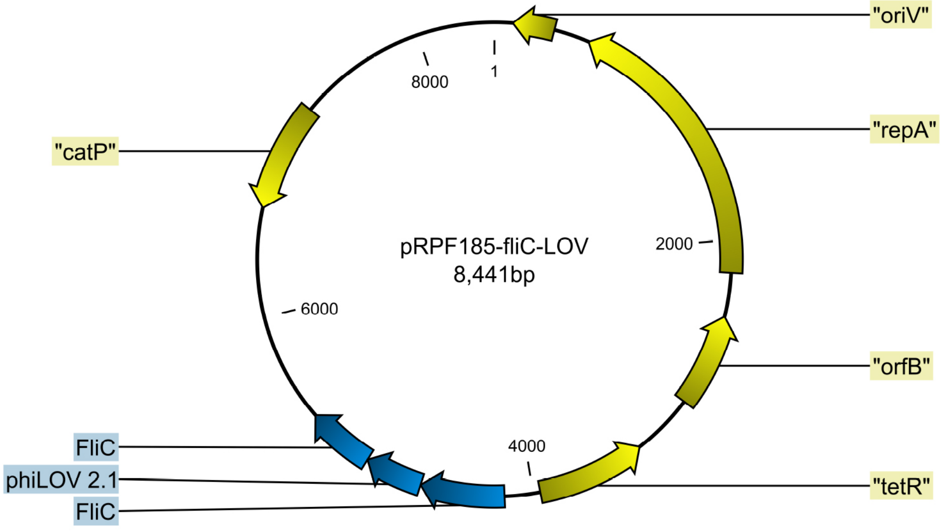

"oriV"
8000
1
"catP"
"repA"
pRPF185-fliC-LOV
8,441bp
2000
6000
"orfB"
FliC
4000
phiLOV 2.1
"tetR"
FliC

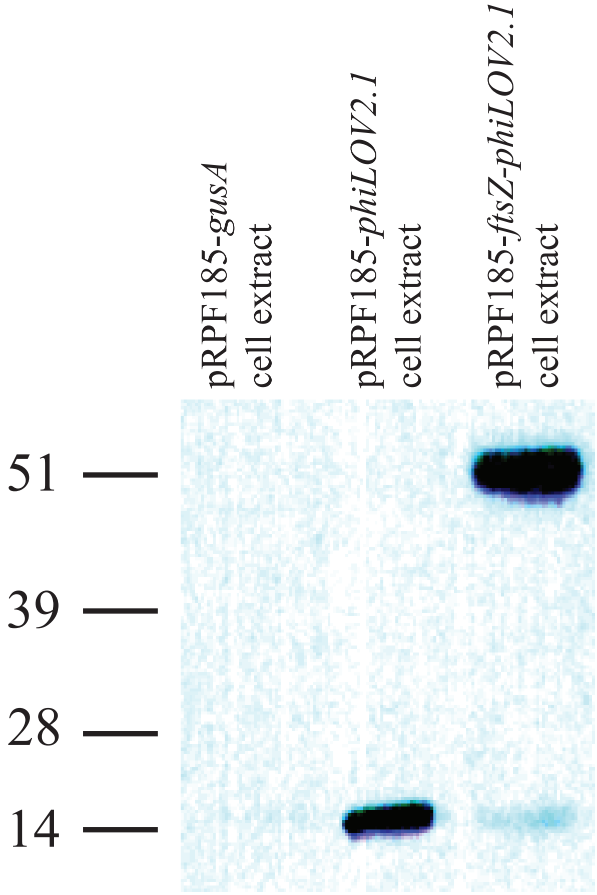
pRPF185-gusA
cell extract
pRPF185-phiLOV2.1
cell extract
pRPF185-ftsZ-phiLOV2.1
cell extract
51
39
28
14

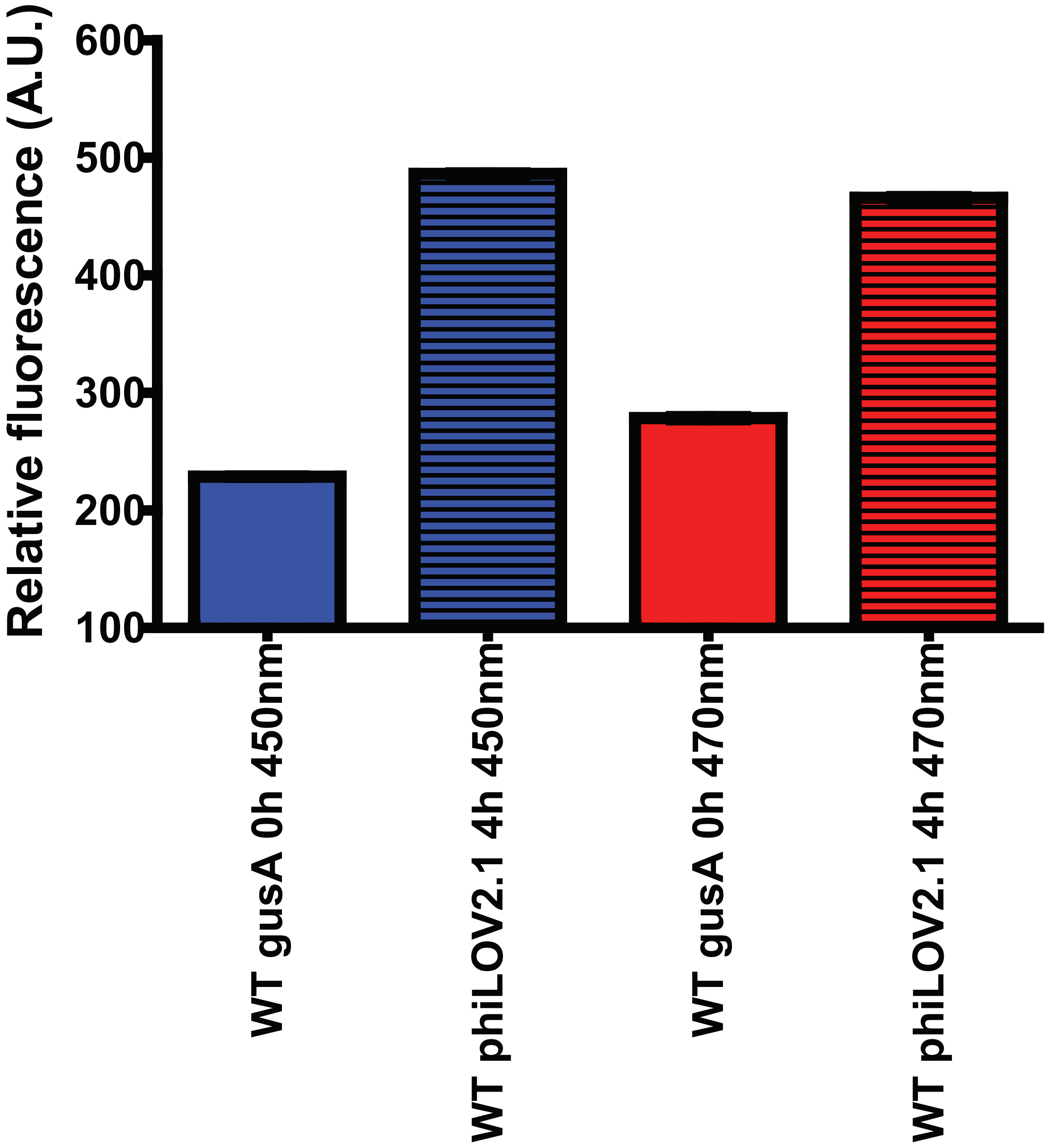
Relative fluorescence (A.U.)
600
500
400
300
200
100
WT gusA 0h 450nm
WT phiLOV2.1 4h 450nm
WT gusA 0h 470nm
WT phiLOV2.1 4h 470nm

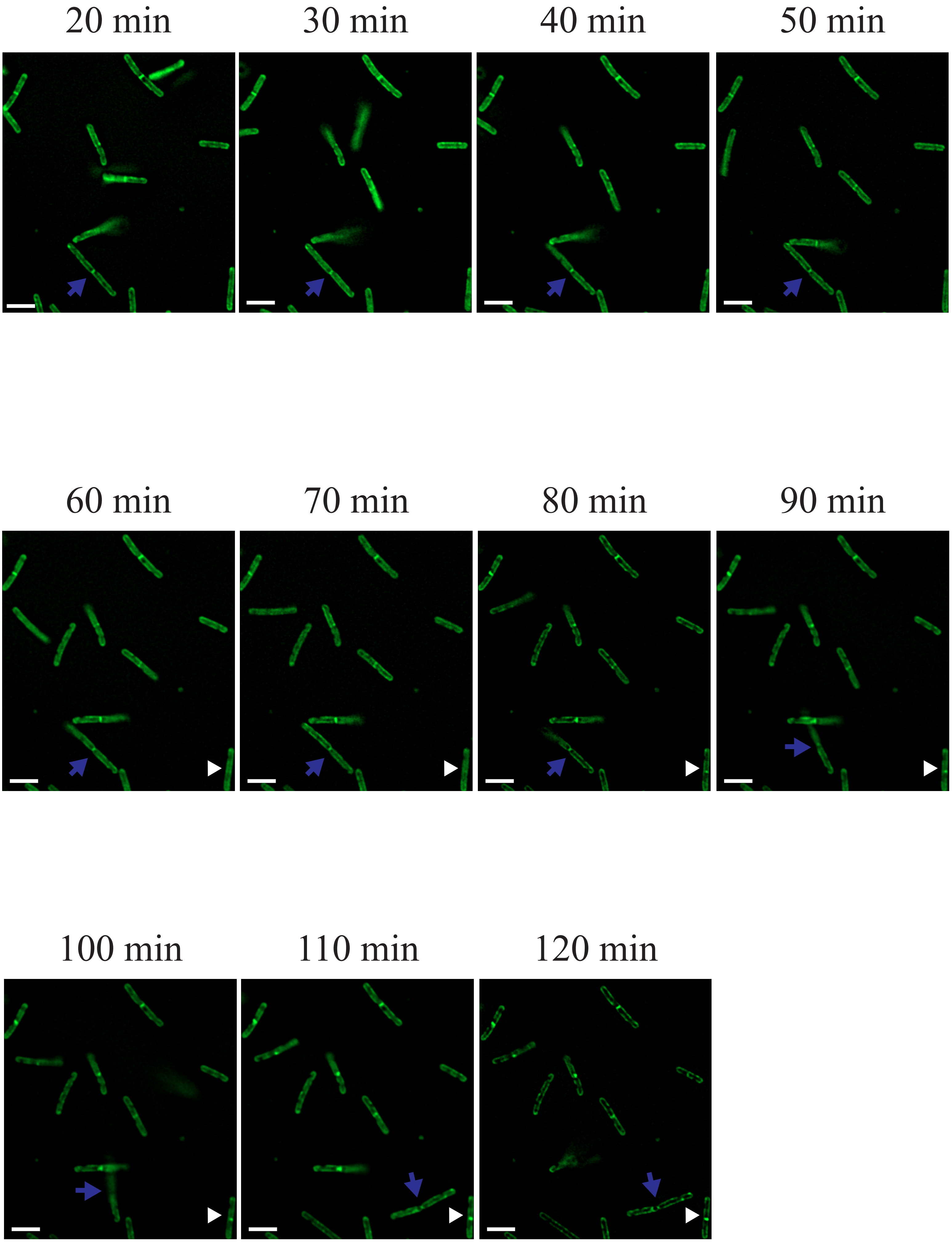
20 min
30 min
40 min
50 min
60 min
70 min
80 min
90 min
100 min
110 min
120 min

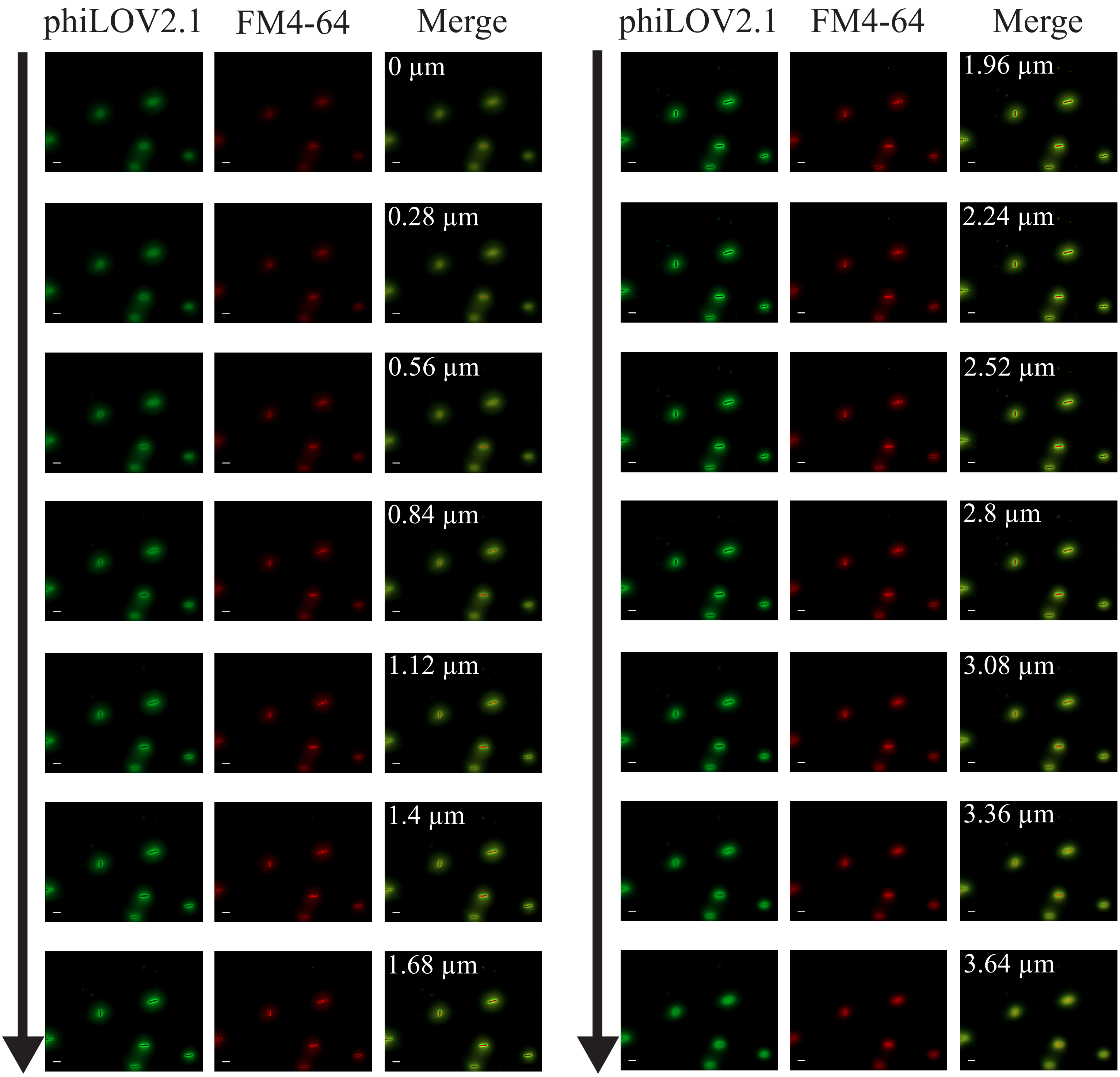

phiLOV2.1
FM4-64
Merge
0 µm
0.28 µm
0.56 µm
0.84 µm
1.12 µm
1.4 µm
1.68 µm
phiLOV2.1
FM4-64
Merge
1.96 µm
2.24 µm
2.52 µm
2.8 µm
3.08 µm
3.36 µm
3.64 µm
